# Unraveling Tempeh through omics: a scoping review of fermentation pathways and functional health benefits

**DOI:** 10.1038/s41538-026-00754-2

**Published:** 2026-02-27

**Authors:** Vira Putri Yarlina, Joel Liano Tandra, Rossi Indiarto, Robi Andoyo, Putri Widyanti Harlina, Nik Hafizah Nik Ubaidillah, Mohd Nizam Lani

**Affiliations:** 1 Department of Food Industrial Technology, Faculty of Agro-Industrial Technology, Universitas Padjadjaran, Bandung, Indonesia; 2 Faculty of Food Science and Agrotechnology, Universiti Malaysia Terengganu, Kuala Nerus, Terengganu, Malaysia

**Keywords:** Biochemistry, Biotechnology, Computational biology and bioinformatics, Microbiology

## Abstract

Tempeh, a traditional Indonesian fermented soybean product, is widely recognized for its functional properties and for containing bioactive compounds produced by microbial fermentation. This study integrates recent advances in multi-omics technologies to elucidate the microbial community dynamics, enzymatic pathways, and metabolite transformation underlying Tempeh’s health-promoting characteristics. A bibliometric analysis of studies published between January 2000 and August 2025, indexed in Scopus, PubMed,Web of Science, and ScienceDirect identified 36 relevant articles that met predefined inclusion criteria. Metagenomic and transcriptomic evidence highlights the important roles of *Rhizopus* species and associated *Lactic acid bacteria* in fermentation, supported by the presence of genes encoding key enzymes such as phytases, amylases, and proteases. Proteomic and peptidomic analyses have further identified bioactive short peptides exhibiting antioxidant and angiotensin-converting enzyme (ACE) inhibitory activities. Metabolomic profiling revealed elevated levels of amino acids, γ-aminobutyric acid (GABA), and isoflavone aglycones, compounds linked to various health benefits. Collectively, these multi-omics insights provide a mechanistic understanding of Tempeh’s functional potential and highlight opportunities for innovation in fermentation optimization and clinical translation. Future integration of standardized fermentation protocols with targeted human studies will be essential to advance Tempeh from a traditional food to a globally recognized functional food product.

## Introduction

Tempeh is a traditional Indonesian fermented food that has been traditionally considered as one of the cultural staples and a cheap source of nutrition^1^. Tempeh is mostly fermented by *Rhizopus* species by solid-state fermentation of soybeans, and more recently by other legumes. It is known to have a firm texture, flavory, and better nutritional value. Other than food use, Tempeh has become a functional food because of its high protein and its increased digestibility and the formation of bioactive compounds generated during fermentation^2,3^. These changes reduce the antinutritional factors and produce peptides, isoflavone aglycones, folate, γ-aminobutyric acid (GABA), all of which are linked with cardiovascular protection, better mineral bioavailability, and gut health^4,5^. Tempeh has a protein and amino acid content of 18-20%. Besides that, Tempeh contains isoflavones (20–60 mg/100 g), folate (72.76 µg/100 g), and GABA (506.68 mg/100 g)^4–6^.

Innovations in new technologies of omics, including genomics, metagenomics, transcriptomics, proteomics, and metabolomics, have created new possibilities in explaining the complexity underlying Tempeh fermentation^6^. Omics systems are very different as compared to the conventional microbiological methods. Classical microbiological methods rely on biochemical analysis and culture-based recognition, and omics are able to give high-resolution mapping of fluxes of metabolites, the expression of functional genes, and microbial communities. Such approaches have always recognized *Rhizopus* molds as the main fermenting organism, with the help of bacterial companions, including *Lactic acid bacteria* (LAB) and *Bacillus* spp. These microbial consortia trigger protein-degrading enzymatic pathways, isoflavone-transforming enzymatic pathways, phytate-degrading enzymatic pathways, and the generation of bioactive metabolites^7,8^. Such a mechanistic perspective puts Tempeh in the context of a fermented product but in a way that allows it to be used as a model system to study the mechanisms by which microbial communities convert food matrices into health-promoting products.

In addition to this, Tempeh possesses several distinctive characteristics that make it a valuable model system for investigating microbiome-food interactions. Compared to other fermented soybean products such as miso, natto, soy sauce, and doenjang, Tempeh features a diverse microbial consortium. In contrast, many of these other products rely on multi-stage mixed-culture fermentation, extended fermentation durations, or single bacterial strains that generate a narrower metabolite profile.

Compared to a large number of other fermented food products, tempeh has unique opportunities regarding multi-omics studies. It is made through a specified starter culture (*Rhizopus* spp.), yet it also grows a heterogeneous bacterial consortium influenced by the raw material, geographic place, as well as processing procedures^9^. It is a reproducible process, culturally embedded, generally consumed, and provides a good basis to study the interplay between microbial ecology, metabolite conversions, as well as host nutrition^10–12^. These features both reveal the significance of Tempeh as a functional food and represent the possibility of its impact on the diversification of the world’s diet and better health of the population.

Though the application of omics in Tempeh studies is on the increase, the existing research is still scattered. Many studies are specific (isoflavone metabolism, peptide profiling, or microbial diversity) and fail to bring together the results of multiple layers of omics. Variations interfere with the comparability of cross-studies in sequencing systems, analysis systems, and fermentation strategies. Despite the numerous descriptions of bioactives in vitro or in vivo models, most of them have not been studied in relation to therapy due to the absence of an evaluation in humans.

This study provides a comprehensive synthesis of omics-based studies on Tempeh fermentation, integrating evidence from genomics, proteomics, and metabolomics to address critical gaps in the current understanding of its functional properties. The purpose of this study is to determine some of the conserved mechanistic pathways, evaluate the potential of bioactive compounds to be translated to humans, and highlight possibilities of clinical validation as well as product innovation. Through the combination of ancient insights and modern results of omics discoveries, the current review demonstrates Tempeh as a novel cultural and scientific model for investigating microbiome-food interactions and the development of next-generation functional foods.

## Results

### Microbiome insights from metagenomics

The microbial succession during fermentation can serve as additional evidence of the changing ecological equilibrium. The first stages are usually characterized by the dominance of LAB, which create favorable pH conditions that enable the proliferation of *Rhizopus*. The fungus predominance in fermentation changes the microbial network and results in the greater production of bioactive metabolites^4^. Table 1 shows the microbiome composition of Tempeh based on the metagenomic studies and the functional implications. When non-soy substrates such as Jack Beans, Cowpeas, and Mung beans are examined using a metagenomic approach, it is found to contain more bacterial diversity, and metabolic products are more unique^9^. The results show that microbial consortia are specific to substrates, which have prospects of product diversification and functional customization of Tempeh.

**Table 1:** Microbiome composition of Tempeh from metagenomic studies and their functional Implications

Microbial group	Source (substrate)	Identification	Representative taxa (from metagenomics)	Functional/Health Implications	References
Bacteria (Soy-based**)**	Soybeans	*Lactic Acid Bacteria* (LAB)	*Lactobacillus plantarum*, *L. fermentum*, *L. casei*	Acidification, flavor development, pathogen inhibition, and probiotic potential	^7,15^
	Soybeans	*Enterococci*	*Enterococcus faecium*, *E. faecalis*	Inhibition of pathogens contributes to microbial stability	^7^
	Soybeans	*Bacillus* spp.	*Bacillus subtilis*, *B. amyloliquefaciens*	Protein hydrolysis; release of peptides; digestibility	^3,55^
	Soybeans (core microbiome)	Mixed LAB and Enterobacteriaceae	*Lactobacillales*, *Enterobacteriales*	Consistently observed across regions; regional diversity	^23^
Fungi (Soy-based)	Soybeans	Molds (starter inoculum)	*Rhizopus oligosporus*, *R. microsporus*	Protein hydrolysis, isoflavone aglycone release, and mineral bioavailability	^3,7^
	Soybeans	Yeasts	*Candida tropicalis*, *Saccharomyces cerevisiae*, *Debaryomyces hansenii*	Aroma, flavor, vitamin production, probiotic potential	^55^
	Soybeans (Over-fermentation, 72 h)	Mixed fungi and bacteria	*Tryblidiopsis sichuanensis*, *Candida* sp., *Lactobacillus* spp., *Klebsiella* spp.	Flavor and aroma; higher diversity in long fermentation	^62^
Bacteria (Non-soy legumes)	Mung bean Tempeh	LAB, *Bacillus* spp.	*Lactobacillus plantarum*, *Bacillus subtilis*	Improved protein digestibility, antioxidant activity	^30^
	Jack bean Tempeh (*Canavalia ensiformis*)	LAB, *Enterobacteriaceae*	*Lactobacillus fermentum*, *Enterobacter cloacae*	Isoflavone biotransformation, mineral bioavailability	^32^
	Jack bean Tempeh (Indonesia)	LAB and *Bacillus* spp.	*L. plantarum*, *Bacillus subtilis*	Enhanced digestibility, bioactive peptide production	^9^
	Winged bean Tempeh (*Psophocarpus tetragonolobus*)	LAB*, Bacillus, Rhizopus*	*Rhizopus microsporus*, *Lactobacillus* spp.	High peptide yield, potential antihypertensive activity	^28^
	Cowpea Tempeh (*Vigna unguiculata*)	LAB and *Bacillus* spp.	*Lactobacillus fermentum*, *Bacillus subtilis*	Improved protein quality, antioxidant peptides	^28^
Fungi (Non-soy legumes)	Mung bean, Jack bean, Winged bean, Cowpea	Molds (starter inoculum)	*Rhizopus oligosporus*, *R. microsporus var. oligosporus*	Essential for fermentation; texture and flavor formation	^9,27,28^

### Microbial enzymes and bioactive metabolites

It is the enzymatic machinery of the Tempeh microbiome that makes it a functional food with a high concentration of bioactive compounds instead of a raw bean. The most significant of these enzymes are regularly identified as glutamate decarboxylases, β-glucosidases, phytases, proteases, and folate biosynthesis pathways. Each serves a specific function in enhancing digestibility, increasing nutrient bioavailability, or generating compounds associated with health benefits—microbial enzymes and bioactive metabolites in soybean and non-soybean Tempeh in Table 2.

**Table 2:** Overview of microbial enzyme production in Tempeh and fermentation types

Enzyme	Microbial sources	Substrate(s)	Process Types	Key metabolites	Functional/Health relevance	References
Proteases /Peptidases	*Rhizopus oligosporus*, *R. microsporus*, *Bacillus subtilis*, LAB (*L. plantarum*)	Soybean, mung bean, jack bean, Cowpea, winged bean	Solid State Fermentation / SSF (Tempeh fermentation)	Small peptides, amino acids	↑ Protein digestibility; ACE-inhibitory peptides; antioxidant activity	^3,30,31^
β-Glucosidases	*Rhizopus* spp., LAB (*L. fermentum*, *L. casei*)	Soybean, jack bean, Cowpea	SSF	Isoflavone aglycones (daidzein, genistein); other polyphenols	↑ Isoflavone bioavailability; antioxidant; estrogenic activity	^28,32^
Phytases	*Rhizopus* spp., *Bacillus* spp.	Soybean, mung bean, jack bean, Cowpea	SSF	Lower inositol phosphates (IP5–IP3)	↑ Mineral bioavailability (Fe, Zn, Ca); ↓ antinutritional phytate	^9,55^
Glutamate decarboxylase	LAB (*L. plantarum*, *L. fermentum*)	Soybean, mung bean	SSF	GABA (γ-aminobutyric acid)	Hypotensive; neuroactive effects	^15,30^
Folate biosynthesis	LAB (*Lactobacillus*, *Enterococcus*)	Soybean, jack bean, cowpea	SSF	Folate (vitamin B9)	Supports one-carbon metabolism; ↓ homocysteine	^7,14,52^
Cobalamin (B_12_-like corrinoids)	*Klebsiella pneumoniae*, *Citrobacter freundii* (debated)	Soybean	SSF	Corrinoid analogues	Claimed B_12_ source but debated bioactivity	^24,25^
Carbohydrases and Esterases	Yeasts (*Saccharomyces cerevisiae*, *Candida tropicalis*)	Soybean, winged bean, Cowpea	SSF	Alcohols, esters, and organic acids	Aroma and flavor development	^28,55^
Colonic fermentation (post-consumption)	Host gut microbiota (SCFA producers)	All legumes	SSF	SCFAs (acetate, propionate, butyrate)	Gut barrier integrity; anti-inflammatory effects; metabolic regulation	^7,55,63^
Fibrinolytic enzyme	*Rhizopus oligosporus* ATCC 6010	Black Soybean		Fibrinolytic protease fractions	↑ Fibrinolytic activity; cardiovascular protection potential	^64^
Digestive proteases (in vitro digestibility assays)	*Rhizopus* spp., mixed consortia	Lotus seed (Nelumbo nucifera), soybean		Improved hydrolysis of proteins → peptides and amino acids	↑ Enzymatic digestibility; enhanced protein quality and bioavailability	^65^

Although soybean Tempeh has been the best characterized, non-soybean legumes, including mung beans, jack beans, cowpeas, and winged beans, have similar enzymatic dynamics, although metabolite profiles are specific to the substrates. The fermentation process relies on the universal enzymatic pillars of proteases, β-glucosidases, and phytases, while decarboxylases and vitamin biosynthesis pathways contribute additional layers of functional value.

### Mechanistic pathways of microbial and metabolites of tempeh bioactives

Complex microbial–substrate interactions during solid-state fermentation are fundamental to the development of Tempeh’s health-promoting properties. *Rhizopus microsporus* (formerly known as *R. oligosporus*), the primary fermenting mold, initiates substrate colonization by secreting a diverse array of hydrolytic enzymes, including lipases, amylases, proteases, and phytases. These enzymes catalyze the degradation of macromolecules into smaller, bioaccessible compounds that act as precursors for the synthesis of functional metabolites. The metabolic profile is further diversified through secondary biochemical transformations mediated by associated bacterial species, particularly LAB and *Bacillus* species, and occasionally by intestinal commensals. The mechanistic pathways linking Tempeh-derived bioactives to their host targets, along with the corresponding evidence tiers, are presented in Table 3.

**Table 3:** Mechanistic pathways of Tempeh bioactives: from metabolites to host targets and evidence tiers

Metabolite/Compound	Main microbial/enzymatic source	Mechanism of action	Host target(s)	Reported functional effect	Omics Evidence	References
Bioactive peptides (ACE-inhibitory, antioxidant)	*Rhizopus oligosporus*, *R. microsporus*, *Bacillus subtilis* (proteases), LAB peptidases	Inhibit ACE; scavenge ROS	ACE; oxidative stress pathways	↓ Blood pressure, ↓ oxidative stress, and improved vascular health	Proteomics, peptidomics, metabolomics	^5,34,66^
Isoflavone aglycones (daidzein, genistein, equol)	*Rhizopus* β-glucosidase; LAB β-glucosidase	Conversion of glycosides → aglycones; ER binding	Estrogen receptors; Nrf2	↑ Antioxidant response, estrogenic modulation, ↓ inflammation	Metabolomics, transcriptomics	^7,32,67^
Lower inositol phosphates (IP5–IP3)	*Rhizopus* phytases; *Bacillus* phytases	Hydrolyze phytate → release bound minerals	Mineral transporters (Fe, Zn, Ca)	↑ Mineral bioavailability; ↓ antinutritional effect	Metagenomics, metabolomics	^55,68^
GABA (γ-aminobutyric acid)	LAB glutamate decarboxylase (*L. plantarum*, *L. fermentum*)	Conversion of glutamate → GABA; neurotransmitter mimic	GABA receptors; vascular smooth muscle	↓ Blood pressure; neuroprotective potential	metabolomics	^15,27,30^
Folate (Vitamin B_9_)	LAB (*Lactobacillus*, *Enterococcus*) biosynthetic pathways	Folate biosynthesis; one-carbon metabolism	Homocysteine pathways	↓ Homocysteine supports DNA methylation	Metagenomics, metabolomics	^7,52,69^
Corrinoids (B_12_-like analogues)	*Klebsiella pneumoniae*, *Citrobacter freundii* (debated)	Corrinoid synthesis	Cobalamin-dependent enzymes	Potential B_12_ replacement (bioactivity debated)	Metagenomics, metabolomics	^25,40,70^
Alcohols, esters, and organic acids	Yeast enzymes (*Saccharomyces cerevisiae*, *Candida tropicalis*)	Carbohydrate fermentation; esterification	Sensory receptors	Flavor, aroma enhancement; potential probiotic effects	metabolomics	^55^
SCFAs (acetate, propionate, butyrate)	Host colonic microbiota fermenting Tempeh fiber/peptides	FFAR2/3 signaling; histone deacetylase inhibition	FFAR2, FFAR3, GPR109A; gut epithelial cells	↑ Gut barrier; ↓ inflammation; metabolic regulation	Metagenomics, metabolomics	^16,33^

### Comparative appraisal of mechanistic pathways in Tempeh fermentation

Comparison of the overall evaluation of the mechanistic pathways (Table 4) also shows that the functionality of Tempeh is largely mediated by a combination of conserved microbial processes. Nevertheless, the power of evidence supporting and translational preparedness differs significantly among metabolites. The relative analysis of the mechanistic pathways highlights the strengths and weaknesses of the available information regarding bioactives derived from Tempeh. Protease-mediated peptide release is a highly reliable and well-reported event, which exhibits a high degree of cross-substrate reproducible peptide release in both non-soy and soy legumes. Proteomic and functional studies give strong in vitro and animal data on inhibition of ACE and antioxidant activity, but have limited human validation^13^. This puts peptide bioactives in either medium or high levels of translational readiness, particularly in incorporation into protein-rich functional meals.

**Table 4:** Comparative appraisal of mechanistic pathways in Tempeh fermentation

Pathway of metabolite	Cross-substrate consistency	Translational readiness	Main levers (to optimize)	Key gaps/caveats	References
Protease → bioactive peptides → ACE/Nrf2	**High** (soy and non-soy)	**Medium–High**	Protease-rich starters; fermentation time/temperature; protein-rich legumes	Dose–response in humans; peptide ID/stability in vivo	^3,5,9^
β-Glucosidase → isoflavone aglycones → ERβ/Nrf2	**Medium–High** (strongest in soy)	**High (soy)** / **Medium (non-soy)**	β-Glucosidase-active strains; control pH; intact seed coats	Tempeh-specific human trials; equol-producer stratification	^7,32,71^
Phytase → lower inositol phosphates → mineral uptake.	**High**	**Medium**	*Rhizopus/Bacillus* phytase activity; pH 4.5–5.5; adequate time	Human absorption/isotope studies; inhibitors (polyphenols) control	^28,55,72^
LAB GAD → GABA → vascular/neuro effects	**Medium** (soy, mung)	**Low–Medium**	GABA-producing LAB; glutamate availability; extended fermentation	Achievable dietary dose; clinical endpoints (BP, stress)	^15,30,59^
LAB → folate (B9) → one-carbon metabolism	**Medium–High**	**Medium**	Folate-producing LAB; oxygen control; minimal heat loss	Human bioavailability; retention during cooking/storage	^58,69^
Enterobacteriaceae corrinoids → “B_12_-like”	Variable	**Very low**	—	Distinguish true cobalamin vs analogues; human bioassays	^25,54,73^
Yeast esters/alcohols → sensory acceptance	**High**	**High (sensory)**	Yeast strain mix; temperature; aeration	Health impact minimal; focus on quality/safety	^55,61^
Colonic SCFAs → FFAR2/3, GPR109A	**High** (all legumes provide residue)	**Medium**	Fiber/protein residue; matrix structure; dose	Tempeh-specific human microbiome/SCFA trials	^7,33,60^

High Consistent across studies, strong evidence (in vitro + animal + some human), Medium Confirmed in vitro/in vivo, no human validation, Low Mechanistic hypothesis only or inconsistent data.

Isoflavone to aglycone conversion catalyzed by β-glucosidase has been ample in numerous substrates and specific molecular links to antioxidant and estrogen receptor-related activities^14^. The pathway shows a high translational preparedness, where the aglycones have high bioavailability and are partially validated by human studies. However, the variations in the case of fermentation conditions and the host responses are still gaps. The phytase pathway is usually witnessed, especially in combination with *Rhizopus* and *Bacillus* fermentation. In vitro and animal research present strong support for the augmented mineral bioavailability, though it is not confirmed in humans.

On the contrary, neuroactive metabolites such as GABA production and B-vitamin production (folate, corrinoids) have reduced translational preparedness. GABA is synthesized by LAB, which is consistent^15^. However, the results differ greatly across the strains, and the evidence is largely preclinical. The pathways of folate biosynthesis are clear. But, its actual role in the dietary intake seems insignificant. The production of corrinoids is highly debatable, as most of the known compounds are analogs of questionable nutritional value. As a result, the path points to low to medium preparedness, which requires a large methodological standardization and studies on human bioavailability.

ESTERs and alcohols produced by the yeast are also sure to add flavor and customer acceptance in sensory aspects. Though health effects are insignificant, translation potential is great, with the quality of the products and marketability. Finally, the short-chain fatty acids (SCFA), which are not synthesized during fermentation, are indirectly promoted by the consumption of Tempeh^16^. Data from animal and indirect human research indicate moderate translational potential for gut health; however, the exact interactions between Tempeh and short-chain fatty acids (SCFAs) remain inadequately characterized.

### Mapping omics approaches in Tempeh research

Omics is a broad term used to describe a set of scientific approaches used to analyze complex biological systems at large scales. These high-throughput technologies enable the comprehensive characterization of biomolecules, such as genes, transcripts, proteins, and metabolites, and capture thousands of molecular entities simultaneously to elucidate their functions and interactions^6,17^. By integrating extensive molecular datasets, omics technologies provide a system-level understanding of biological processes, moving beyond the limitations of studying individual components in isolation.

In the context of fermentation science, omics methodologies, particularly genomics, metagenomics, transcriptomics, proteomics, and metabolomics, have become indispensable for exploring microbial community dynamics and functional pathways. Applied to Tempeh research, these methodologies have enabled researchers to map patterns of gene expression, identify key enzymes involved in substrate transformation, and monitor changes in metabolic pathways throughout fermentation.

Through multi-layered analytical integration, omics-based research provides profound insights into the molecular and microbial interactions that govern Tempeh fermentation. Such insights facilitate the optimization of fermentation conditions, the enhancement of product functionality, and the improvement of nutritional profiles in fermented foods.

The study synthesizes findings from multiple omics platforms to characterize microbial activity, metabolic networks, and the formation of bioactive compounds during Tempeh fermentation. The combined evidence highlights the potential of omics-driven approaches to advance both fundamental understanding and applied innovation in functional food development. A summary of omics methodologies, analytical platforms, major findings, and current limitations in Tempeh studies is provided in Table 5.

**Table 5:** Omics methods, platforms, findings, and limitations in included Tempeh studies

Omics Type	Platforms Used	Targets Analyzed	Findings	Limitations	References
Metagenomics	16S rRNA sequencing; ITS sequencing; Shotgun metagenomics	Microbial communities; Functional gene clusters	*Rhizopus microsporus/oligosporus* consistently appears as the dominant fungus, a stable group of *Lactic Acid Bacteria* (*Lactobacillus plantarum*, *L. fermentum*, and *Enterococcus)*. Shotgun analyses reveal genes linked to β-glucosidase, protease, and phytase activity.	Limited sequencing depth, primer bias, and uneven metadata reporting reduce comparability. Functional annotation is often incomplete, and few studies include time-series sampling.	^7,9,10,12,21^
Metabolomics (Non-Volatile)	LC–HRMS; LC–MS/MS; UHPLC-QTOF MS; HPLC; NMR	Isoflavones, amino acids, organic acids, and polyphenols	Fermentation increases levels of daidzein and genistein aglycones. Free amino acids and antioxidant compounds rise significantly in the final product.	Many datasets are semi-quantitative. Extraction methods differ widely. Several studies lack standardized metabolite libraries for identification.	^8,74,75^
Metabolomics (Volatile)	GC–MS; GC×GC–TOF MS; HS-SPME–GC–MS	Aroma volatiles (alcohols, esters, ketones, organic acids)	Volatile profiles vary with substrate and fermentation temperature. Key aroma compounds originate from *Rhizopus* and yeast metabolism.	Volatile loss during sampling and incomplete annotation are common challenges.	^76,77^
Proteomics/Peptidomics	LC–MS/MS; MALDI-TOF MS; 2D-PAGE	Proteases; Peptides	Fermentation generates peptides with ACE-inhibitory, antioxidant, and other bioactive properties. *Rhizopus* proteolysis is the primary mechanism, and substrate selection influences peptide diversity.	Protein extraction is complex, peptide databases are limited, and reproducibility across studies is low.	^5,78^
Genomics	Whole-genome sequencing	*Rhizopus* starter strains; LAB isolates	Genomes reveal clusters encoding proteases, β-glucosidases, and lipases. Genomic differences between strains correspond to functional variation in fermentation.	Very few genomes are available, limiting broader comparisons and functional validation.	^79,80^
Transcriptomics	RNA-Seq	Gene expression patterns	Early fermentation shows strong expression of *Rhizopus* proteases and carbohydrate-active enzymes.	Single study; limited scope; no replication or comparison across substrates.	^81^
Multi-Omics	Metagenomics + metabolomics; Proteomics + metabolomics	Integration of microbial composition, enzymes, and metabolite profiles	The complex relationship between geographical origin, packaging materials, production environments, and microbial community dynamics in Tempeh. Protease activity correlates with bioactive peptide formation. Multi-layer data reveal clear microbe–enzyme–metabolite relationships.	Sampling is often asynchronous, and bioinformatic workflows vary, making integration difficult.	^82^
Bioinformatics Tools	Software and pipelines used for preprocessing, feature extraction, and annotation.	- *QIIME2* - *HUMAnN3* - *XCMS* - *MetaboAnalyst*	Sequence clustering and taxonomy Functional pathway prediction metabolite peak alignment Statistical modeling	-	^83^
Statistical Analysis	Analytical methods are used to explore and interpret the high-dimensional data sets.	- PCA, PLS-DA - Spearman correlation - t-tests/ANOVA	Multivariate pattern recognition **Spearman correlation** between taxa and metabolitesIdentifying significant differences	-	^74,77^
Database Integration	Mapping microbial genes and metabolites: biological functions and pathways using reference databases.	- Taxonomic - Functional -Metabolite Identification:	SILVA, Greengenes KEGG Orthology (KO), MetaCyc, UniRef HMDB, MassBank, METLIN	-	^84–86^

## Discussion

**Fig. 1:**
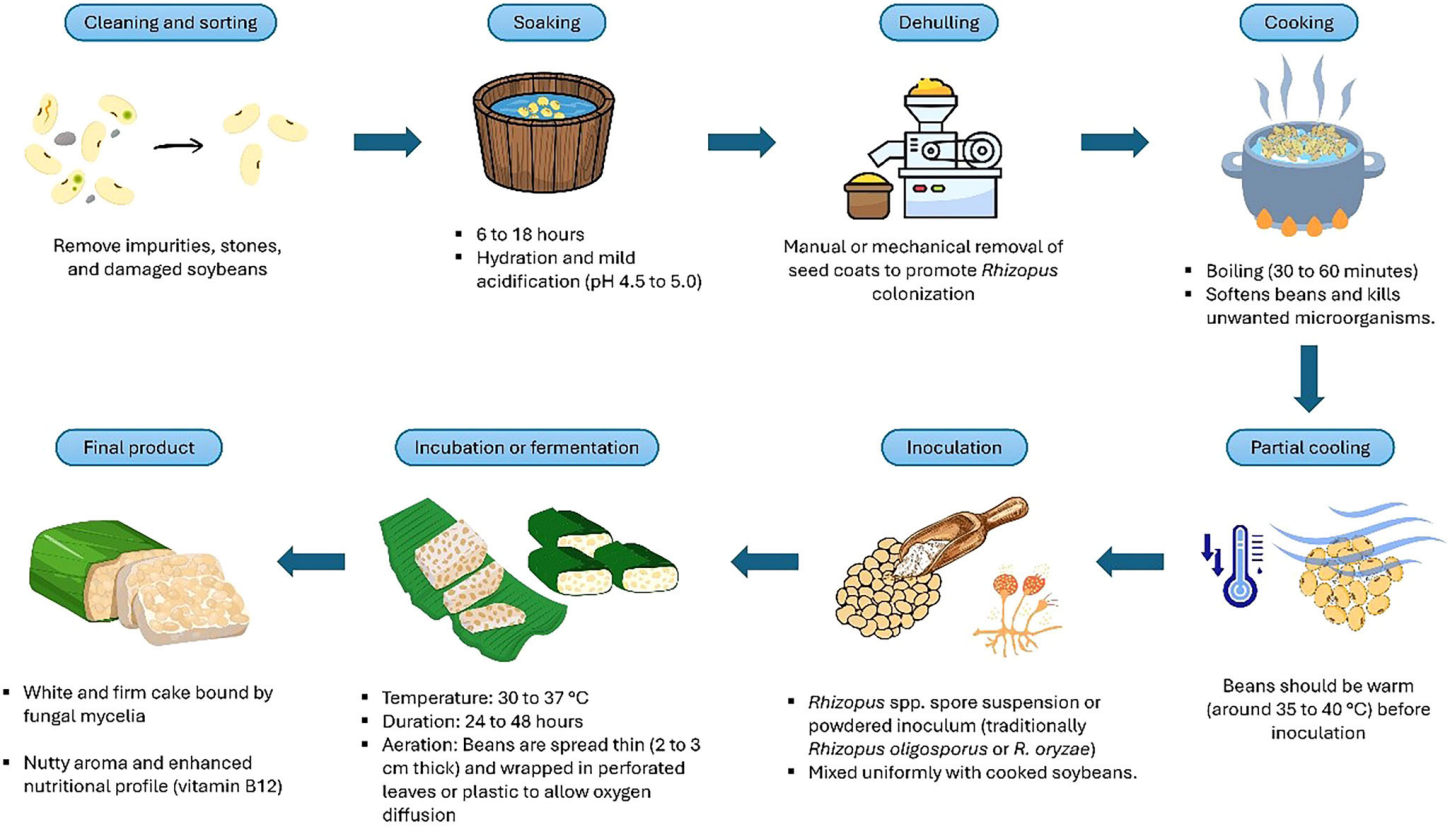
The Traditional Indonesian Tempeh Production Process.

Tempeh is a fermented food in Indonesia, with a usual process that includes soaking, dehulling, cooking, inoculation with starter culture (Raprima merk), and solid-state fermentation at specified temperatures and for defined periods, producing a dense, cake-like texture and a distinctive nutty flavor. Figure 1 presents the usual process of Tempeh production in Indonesia.

Omics technologies have enabled the study of microbial communities, physiology, bioactive peptides, metabolic pathways, and the resulting functional compounds. Metagenomics and transcriptomics enabled mapping microbial succession and functional gene expression. Proteomics has enabled the mapping of proteins and bioactive peptides, providing mechanistic insights into protein interactions that underlie health-related functions and biological processes.

The microbial environment present in Tempeh ensures a strict balance between fungi and bacteria, where each population works with specific roles in converting legumes into a nutritionally enriched, bioactive food product. The most common fungus is *Rhizopus oligosporus* (90%), whereas *Lactobacillus* spp. (10–45%), *Bacillus* spp. (5–30%), and *Enterococcus* spp. (2–15%). They are the most frequent bacterial species.

Metagenomics has greatly contributed to a better understanding of the microbial ecosystem of Tempeh^9^. As compared to the conventional culture-based techniques that do not capture diversity, sequencing techniques show a complex consortium of fungi and bacteria that work in a synergistic way to promote fermentation. Besides affecting the safety and sensory properties of Tempeh, the microbial community also synthesizes bioactive compounds of interest to human health. *Rhizopus oligosporus* and *R. oryzae* are always found to be the most common fungi in these spaces, as per the metagenomic studies. These species produce extracellular enzymes, such as proteases, lipases, and phytases, that promote protein digestibility, degrade lipids, and antinutritional factors such as phytate^2,3^.

Bacterial consortia and fungi have a great role in the determination of fermentation products. According to high-throughput sequencing, *Rhizopus* is often co-occurring with LAB (*Lactobacillus*), spore-forming *Bacillus*, and *enterococci*. Bacteria also help in the creation of flavor, release antimicrobial peptides, and stabilize the microbial community through the production of organic acids that suppress the growth of unwanted organisms^7,18^. *Enterococcus* strains that produce bacteriocins have been discovered, highlighting the possible role of Tempeh in food safety. Functional genes and pathways favoring bioactivity are emphasized by metagenomic information. Isoflavone glycosides are converted to aglycones by genes that encode β-glucosidases, which are more potent antioxidants and estrogens^19^. An association between genes coded to phytase and reduced phytate concentrations and enhanced mineral bioavailability exists. Additional potentially beneficial neuromodulatory benefits of Tempeh intake are indicated by other avenues of GABA biosynthesis.

*Rhizopus oligosporus* and *R. microsporus* are filamentous fungi that are at the core of this ecosystem, and which are always known to be the most common molds in Tempeh fermentation^20^. The high rate of mycelial growth joins soybeans and other legumes in the tight cake-like Tempeh that allows numerous enzymatic actions. These fungi produce proteases, amylases, lipases, and phytases that break down complex macromolecules to smaller and more accessible ones. The result is a product with an increased protein digestibility, a better mineral availability, as well as increased concentrations of bioactive metabolites, such as isoflavone aglycones^3,21^.

The most ubiquitous of the dynamic bacterial consortia that envelops this fungal center is LAB, including *Lactobacillus plantarum* and *Lactobacillus fermentum*^22^. These microorganisms acidify the medium, release bacteriocins which prevent the growth of spoilage microorganisms, and these organisms also work together with *Rhizopus* to ensure a stable fermentation process. *Bacillus* species, in particular, *B. subtilis*, are of significance. Proteins are used as proteases and amylases; the difference is in the process of hydrolysis, which results in small peptides and a more complex flavor. *Enterococcus* species have been observed in various studies and could possibly be involved in proteolysis and acidification, but the presence of this species is usually treated with caution since they may be opportunistic pathogens^7,23^. *Klebsiella pneumoniae* is considered the most controversial participant of the Tempeh microbiome. This study was preceded by a study that employed the application of culture-based identification, which suggested that *Klebsiella* might be instrumental in the production of vitamin B_12_ in Tempeh and hence improved the value of the product to the vegetarian diets^24^. Recent studies have cast doubt on the original purpose of the identified corrinoid compounds as bioactive B_12_ or mere analogues that have little nutritional use^25^. This discussion brings back the reconsideration of the historical microbiological discoveries by modern molecular techniques.

The use of the omics technologies has changed the knowledge of the Tempeh microbiome at its core. In the past, the study has been based mainly on the cultivation technique, thus leaving behind those that are unculturable or those whose concentration is low. Conversely, more detailed information on bacterial and fungal community structures has been obtained in recent times with the use of modern 16S rRNA and ITS sequencing tools. It has been established through studies that *Rhizopus* molds and LAB are predominant, but it is also possible that minority taxa may also influence flavor, safety, or the production of certain metabolites^7,23^. Besides community mapping, metagenomics has also determined particular microbes contained in functional groupings of genes, including protease, phytase, and β-glucosidase activities. The use of the metatranscriptomic techniques gives very useful information in that the presence of genes is not the only information, but the active involvement of the genes during the fermentation process. The experiments show that *Rhizopus* proteases and phytases reach their maximum activity in the first few hours of fermentation. On the contrary, bacterial carbohydrate-active enzymes are more common at later stages, in accordance with the acidification and flavor maturation process^26^. This time-dependent activity suggests that Tempeh fermentation is a dynamic process that is defined by a series of microbial activities, that is, fungi and bacteria complement each other in their roles at various stages.

The findings indicate that the Tempeh microbiome is stable and malleable. The regular activity of *Rhizopus* molds is necessary. The diversity of the bacteria depends on the nature of the raw material (soybeans and other legumes), geographic practices, and the period of fermentation. This flexibility of the legumes explains the common microbial structure of Mung Bean, Jack Bean, Winged Bean, and Cowpea Tempeh, and unique differences in bacterial count and enzymatic functions^9,27,28^. Indonesian researches on Jack Bean Tempeh report the importance of *Lactobacillus* and *Bacillus* in creating peptides that have bioactive potential^9^. Conversely, African studies regarding the role of winged bean and cowpea Tempeh have shown that the latter can be used to generate LAB-rich fermentations that generate antioxidant and antihypertensive peptides.

The activity of glutamate decarboxylase in *Lactobacillus plantarum* and *Lactobacillus fermentum* makes it possible to produce a bioactive compound, γ-aminobutyric acid (GABA), which has neuroactive and hypotensive properties. Though the concentrations are moderate compared with specialist GABA-enriched foods, studies have shown that soybean has been significantly increased in mung bean Tempeh^15,27^. The data show that the fermentation environment or nutritional supplementation of substrates can be optimally adjusted to boost the GABA synthesis, thereby increasing the functional food profile of Tempeh.

Another important contribution is made by the mechanisms of folate production, which are mainly generated by *Lactobacillus* and *Enterococcus* and mediated by LAB. Rezac et al.^7^ confirm that the synthesis of folates in soybean Tempeh is critical in reducing the level of the homocysteine amino acid and enhancing the metabolism of one-carbon. Also, initial tests show folate accumulation in Cowpea and Jack Bean Tempeh, which can confirm the idea that LAB activities are preserved in substrates^9,29^. Vitamin B_12_ or cobalamin-like compounds still remain disputable. Recent findings in the field of omics suggest that the *Klebsiella pneumoniae* and *Citrobacter freundii* are often involved in the production of corrinoid analogs of low bioactivity, though the literature on the topic suggests that such bacteria are involved in B_12_ production^14,25^. Such a difference is an indication of the need to integrate metabolomics and bioassays in order to authenticate nutritional claims.

The microbiome of Tempeh is described as a fungi-bacteria consortium, in which *Rhizopus* molds are used to make the primary transformation, and bacteria are used to optimize nutritional and sensory characteristics. Omics-driven research is providing mechanistic information elucidating long-standing observations and making the manipulation of substrates and starter cultures less deliberate. These strategies can allow optimizing Tempeh to taste and texture and use it as a microbiome-driven food whose relevance goes beyond its Indonesian setting.

Enzyme of Tempeh production give health benefit and significant importance, such as proteases produced by *Rhizopus oligosporus*, *R*. *microsporus*, and co-fermenting bacteria, including *Bacillus subtilis* and LAB. In addition to increasing nutritional value, they hydrolyze storage proteins into smaller peptides and amino acids, producing bioactive peptides with antihypertensive and antioxidant qualities. While similar proteolytic activity has been observed in non-soybean substrates like mung beans, jack beans, and cowpea, although the peptide profile varies depending on the legume matrix, multiple in vitro studies have confirmed the presence of ACE-inhibitory peptides in soybean Tempeh^30,31^. Protease activity is retained, but a variety of substrates introduces novel peptide repertoires with possibly different bioactivities, according to this theory.

Another characteristic of Tempeh fermentation is the activity of β-glucosidases, which are primarily found in LAB and *Rhizopus* molds^32^, these enzymes hydrolyze isoflavone glycosides in soybeans to produce aglycones such as genistein and daidzein, which exhibit more pronounced estrogenic and antioxidant actions and better intestinal absorption. This conversion exemplifies the microbiome’s role in enhancing bioactive compounds in fermented foods. Non-soy legumes, including jack bean and cowpea, exhibit lower isoflavone levels but still demonstrate β-glucosidase activity, which has been associated with enhanced polyphenol availability and antioxidant effects^28^. Consequently, β-glucosidase activity is a conserved function across various substrates, although the specific metabolites vary according to the phytochemical profile of the legume.

Phytase activity, mainly associated with *Rhizopus* and *Bacillus* species, is essential for degrading phytate, the primary antinutrient found in legumes. Lower inositol phosphates improve the bioavailability of calcium, zinc, and iron by reducing mineral chelation^3,4^. This has been shown in both soybean and non-soybean Tempeh. The importance of this enzymatic route in diversifying Tempeh beyond soy is highlighted by the correlation between increased mineral bioaccessibility and phytase activity in jack bean Tempeh^20^.

The host’s intestinal microbiota fermentation byproducts must not be disregarded. Tempeh provides substrates, including refractory peptides and dietary fiber, that are fermented by microbes in the human gastrointestinal tract, producing short-chain fatty acids (SCFAs) such as acetate, propionate, and butyrate. Mediators of intestinal barrier integrity, inflammatory regulation, and metabolic health are known as these short-chain fatty acids^4,7,33^. The impact of the Tempeh microbiota is not restricted to the fermentation chamber, and it also affects the gastrointestinal ecology of the host.

The mechanistic pathways of Tempeh result by metabolomics analysis. Some of metabolite found on tempeh production, such as bioactive peptides, isoflavone aglycones, IP5-IP3, GABA, Folate, B_12_, Organic acids, and SCFAs. *Rhizopus oligosporus* and *R. microsporus* release proteases that hydrolyze legume proteins into smaller peptides, while *Bacillus subtilis* and LAB peptidases enhance this process. These peptides have consistently demonstrated the ability to block angiotensin-converting enzyme (ACE) in vitro, indicating a hypotensive action^3,34^. Animal studies have demonstrated a decrease in systolic blood pressure in hypertensive rat models treated with Tempeh hydrolysates, as well as improvements in oxidative stress indicators, including malondialdehyde levels and glutathione activity^35^.

Non-soy legumes, including Jack Bean, Mung Bean, and Cowpea, exhibit comparable proteolytic activity; however, the peptide sequences vary due to differences in substrates^9,36,37^. This indicates that ACE-inhibitory capacity is a conserved result, although the variety of peptides may expand the functional range.

**Fig. 2:**
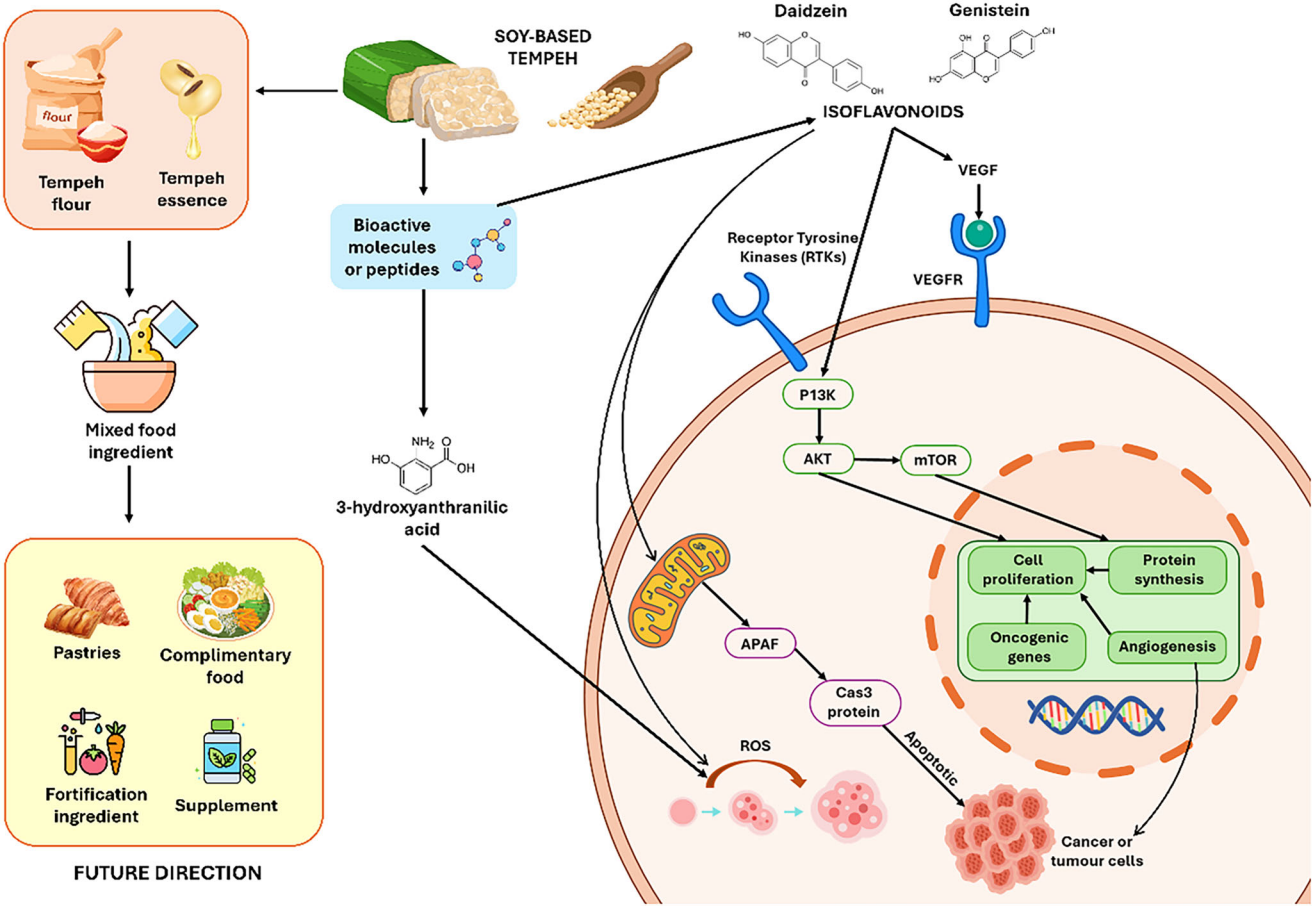
Pathway of Isoflavones Metabolite of Tempe.

Isoflavones represent a significant characteristic of Tempeh’s bioactivity. In unfermented soybeans, isoflavones exist as glycosides, which have low absorption rates. During fermentation, β-glucosidases from *Rhizopus* and LAB hydrolyze them into aglycones, such as daidzein and genistein, which are more efficiently absorbed in the intestine. These aglycones can bind to estrogen receptors and activate Nrf_2_, thereby increasing the production of antioxidant enzymes^7,38,39^. Animal research has shown decreases in oxidative damage and lipid peroxidation, but human studies on soy isoflavones—albeit not unique to Tempeh—indicate enhancements in vascular function and alleviation of menopausal symptoms^40^. When compared to unfermented soybeans, soybean tempeh usually contains 20–60 mg total isoflavones per 100 g, with genistein and daidzein aglycones increasing by roughly 2–4 times. The pathway of isoflavone metabolites of Tempeh can be shown in Fig. 2.

Tempeh made from soybeans is widely recognized as a functional food due to its high content of bioactive compounds, including peptides and isoflavonoids such as daidzein and genistein. Fermentation is essential for making these molecules more bioavailable because it breaks down glycosidic bonds, which raises their aglycone content and biological activity^41,42^. The compounds are key to cellular pathways that control oxidative balance, immune response, and cancer prevention.

Figure 2 illustrates how bioactive compounds in soybeans used to make Tempeh can affect various molecular mechanisms, particularly those involved in tumor growth. Peptides are formed during tempeh fermentation independently of isoflavone metabolism, as a result of *Rhizopus* protease-mediated hydrolysis of soybean proteins. These peptides are neither substrates nor intermediates in the transformation of isoflavone glycosides (daidzin, genistin) into aglycones (daidzein, genistein).

Isoflavonoids interact with receptor tyrosine kinases and vascular endothelial growth factor receptors (VEGFRs), which are essential for controlling angiogenesis and cell growth. Genistein blocks the PI3K/AKT/mTOR signaling pathway, leading to lower expression of oncogenic genes, reduced angiogenesis, and inhibited tumor cell development^43,44^. Daidzein has similar effects, but they are usually weaker. Daidzein interacts with genistein to alter the downstream targets of these pathways^45^. Tempeh-derived isoflavonoids may lower the risk of cancer through dietary intervention by slowing the PI3K/AKT/mTOR cascade.

The 3-hydroxyanthranilic acid, a tryptophan metabolite, is essential for controlling oxidative stress. High levels of this metabolite can control mitochondrial pathways, which can cause the release of reactive oxygen species (ROS) that start apoptosis by turning on the APAF and Caspase-3 proteins^46,47^.

Figure 2 shows that Tempeh flour and essence can be used in a variety of product formulations, such as pastries, complementary foods, fortified ingredients, and dietary supplements. These applications are in line with the rising customer needs of healthy functional foods that prevent disease, as well as are healthy^48^. Also, the inclusion of Tempeh-based foods in the child nutrition programs can help alleviate malnutrition and stunting, particularly in regions where access to animal-based proteins is limited^9^.

Legumes contain phytate, which is an important antinutritional factor, because it complexes important minerals. The phytates are broken up by phytases of *Rhizopus* and *Bacillus* into smaller inositol phosphates, releasing bound minerals. Different in vitro digestive systems show increased solubility of iron and zinc in fermentation^12,38^, and animal experiments show that rats fed on Tempeh have a higher hemoglobin regeneration capacity compared to nonfermented beans^49^. This process does not apply just to soy but also to other legumes, including the jack bean, where phytase activity correlates with an increase in the bioaccessibility of iron and zinc. However, there are no human trials, and above all, without direct absorption or isotope tracer studies, no conclusions can be made about mineral bioavailability. The route is physiologically authentic and indicates one of the most nutritionally important outcomes of Tempeh fermentation.

The accumulation of γ-aminobutyric acid (GABA) in Tempeh is due mainly to microbial glutamate decarboxylase (GAD) activity, a process observed in both soybean-based and non-soy legume Tempeh. During fermentation, LAB, especially *Lactobacillus plantarum* or *L. fermentum*, catalyze the conversion of glutamate to GABA, and these effects increase GABA levels in soybean Tempeh by large margins relative to unfermented beans^15,27^. In addition to LAB species such as *Enterococcus*, *Lactococcus*, *Leuconostoc*, and *Streptococcus*, certain *Bacillus* and yeast strains possessing GAD-encoding genes have been associated with GABA synthesis in fermented foods. Although these taxa generally occur at lower abundances than LAB in Tempeh fermentations, their collective genetic potential suggests that GABA production is a community-level metabolic function rather than being limited to particular LAB strains.

GABA production in Tempeh is influenced by the type of substrate used. Soybeans and non-soy legumes differ in their initial amino acid composition, particularly in glutamate concentrations, and in the extent of proteolysis occurring during *Rhizopus*-mediated fermentation. Substrates with elevated intrinsic glutamate concentrations or those subjected to enhanced proteolysis yield a larger free glutamate reservoir, thereby facilitating greater GABA biosynthesis. The different legumes (mung beans, Jack beans, and cowpeas) can exhibit varying levels of GABA accumulation during fermentation, reflecting differences in their protein structures and amino acid profiles that have different metabolic potentials for different substrates.

In vitro experiments validate the production, while animal models demonstrate a decrease in blood pressure following the consumption of GABA-enriched fermented food^15,30^. Comparable findings have been reported for Mung Bean Tempeh, indicating that substrate composition affects glutamate availability and, consequently, GABA yield^50^.

Folate biosynthesis during tempeh fermentation with LAB, as confirmed by both culture-dependent assays and genetic analysis that anticipate folate biosynthesis pathways^51^. Folate is involved in the one-carbon metabolism, DNA synthesis, and homocysteine regulation. The in vitro tests show an increase in folate level following fermentation, but the rodent tests confirm its bioavailability. Essentially, non-soy legumes such as Jack Bean and Cowpea are folate-enriched^52,53^. The finding is promising to the population groups that are at risk of folate deficiency. But human information is extremely limited, and there is no clear indication on whether folate, as a by-product of Tempeh, is of significant benefit to nutritional adequacy in real-life diets. Thus, despite the clear evidence of the microbial potential, the nutritional effect requires human confirmation.

One of the most controversial issues of the nutritional profile of Tempeh is the possible contribution of vitamin B_12_ (Cobalamin). Preliminary studies proposed that corrinoid compounds produced by bacterial consortia during fermentation could possibly be found in quantifiable amounts in Tempeh^24,54^. These results created a lot of interest, particularly among the vegetarian and vegan communities, because B_12_ is usually found in non-vegetarian diets.

The majority of the corrinoids found in Tempeh are not biologically active versions of vitamin B_12_, but are analogs that can either not bind as a cofactor or may interfere with B_12_ metabolism^25^. These analogues can affect the B_12_ assays, resulting in false-positive results and overestimation of the true nutritional value.

Furthermore, the microbial pathways that generate active cobalamin are inadequately characterized, as *Rhizopus* does not produce B_12_, and the bacterial associates involved, such as *Klebsiella pneumoniae* and *Propionibacterium freudenreichii*, are sporadically found in natural fermentation.

Yeasts such as *Saccharomyces cerevisiae* and *Candida tropicalis* contribute carbohydrases and esterases, generating alcohols, esters, and organic acids. These compounds are primarily responsible for Tempeh’s aroma and flavor but may also contribute minor probiotic or antioxidant effects^4,55^. Their contribution to consumer acceptance cannot be overlooked since their direct health effects are insignificant when compared with those of peptides or isoflavones, but their cultural and dietary significance cannot be ignored to make Tempeh culturally and dietarily relevant.

The intestinal microbiota is able to produce short-chain fatty acids (SCFA). Acetate, propionate, and butyrate are produced by the intestinal microbiota using partially hydrolyzed substrates, dietary fiber, and resistant proteins introduced via Tempeh intake. These SCFAs communicate with FFAR2/3 receptors and GPR109A, which improves gut barrier activity, alters immunological reactions, and controls metabolism^16,33^. These effects are regularly supported by animal research, but the human results are obtained as a result of fiber and prebiotic studies and not the specific Tempeh intervention. This is the case of fermented foods, which indirectly influence the host physiology through the changes in gut flora.

Tempeh plays a greater role in cardiovascular health promotion. Protein-derived peptides of *Rhizopus, Bacillus*, and LAB exhibit considerable ACE-inhibitory and antioxidant effects, which is why the proposal of the mechanism of antihypertensive effects is possible. In vitro and animal experiments have indicated a decrease in blood pressure and oxidative stress with exposure to Tempeh-derived peptides^3,56^. This pathway is also preserved in non-soy substrates^9^ as indicated by recent studies on a Jack Bean Tempeh.

Isoflavone aglycones generated via β-glucosidase activity enhance this cardiovascular potential. The fermentation of Tempeh enhances antioxidant activity and facilitates estrogen receptor–mediated vascular benefits by hydrolyzing glycosylated isoflavones into their more bioavailable aglycone forms^40,57^. Clinical evidence regarding soy isoflavones indicates enhanced vascular elasticity and alleviation of menopausal symptoms^14^. The findings support the use of Tempeh peptides and isoflavones in the formulation of functional supplements, peptide-rich protein powders, and nutraceutical products designed for populations at risk of hypertension, cardiovascular disease, and oxidative stress.

Tempeh can play a major role in alleviating the shortage of micronutrients, which has continued to be a challenge in plant-based diets. Phytic acid is decreased by the production of phytases by *Rhizopus* and *Bacillus*, which liberate bound minerals and increase the bioavailability of iron, zinc, and calcium. Models of in vitro digestion and animal research studies provide evidence that the solubility of minerals and hemoglobin regeneration improve significantly with the intake of fermented legumes^28,49^. LAB is also able to augment folate fortification throughout the fermentation procedure, hence elevating Vitamin B_9_ in Tempeh^7,58^. Folate fortification is highly important in the one-carbon metabolism and homocysteine regulation, and it is important to note that it has critical consequences on maternal and child health. The pathways reflect how Tempeh can be used as a delivery system of functional flour, fortified noodles, school-feeding snacks, and supplements to support the objectives of pregnant women in high-stunting, anemia, and neural tube defect regions.

**Fig. 3:**
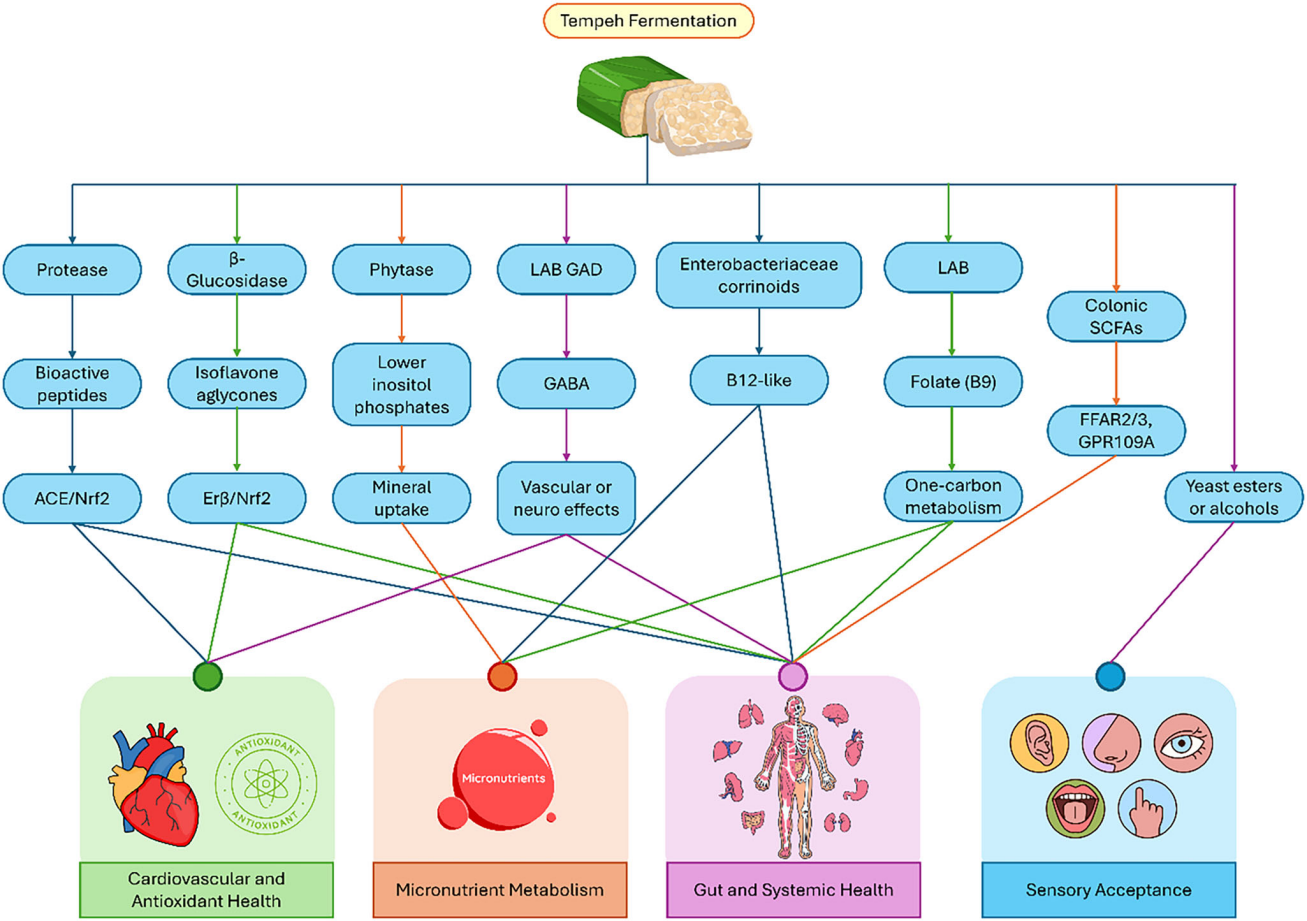
Metabolites of Tempeh Generated in Human Health.

Legumes are transformed into Tempeh by fermentation of the microorganisms to add value and produce a variety of bioactive compounds that have established health effects. These pathways are supported by in vitro tests, animal experiments, and, in a few cases, small-scale human experiments. Such results make Tempeh one of the emerging functional food platforms, which includes both traditional and innovative uses. Figure 3 demonstrates the metabolites of Tempeh produced in human health.

Figure 3 shows metabolites of tempeh that are generated in human health, including cardiovascular health, micronutrient metabolism, gut and systemic health, and sensory acceptance. Along with cardiovascular and nutritional actions, Tempeh bioactives can be involved in the influence on the neurological functionality and stress regulation. Although synthesized in small quantities, LAB-generated GABA has been associated with blood pressure decrease and sedation in the animal models^30,59^. There are no clinical studies that have directly evaluated GABA in Tempeh, but the optimization of starter cultures and fermentation could potentially increase GABA production, and in the future, GABA-enriched drinks, teas, or nutraceuticals can be developed to relieve stress and manage mild hypertension. It is possible that isoflavones and antioxidant peptides can have neuroprotective effects via Nrf2-mediated antioxidant responses, suggesting that Tempeh could serve as a potential functional food for cognitive health; however, additional evidence is necessary.

The other essential field of functional implementation is gut health and microbiome modulation. In addition to the direct contribution of the probiotics LAB, Tempeh contains precursors, including fiber, resistant proteins, and partially fermented peptides, which are fermented in the colon to produce SCFAs, including acetate, propionate, and butyrate. These metabolites exert their action through FFAR2/3 and GPR109A receptors and increase gut barrier integrity, regulate immune responses, and control metabolic pathways^33,60^. Although the benefits of SCFA have been well demonstrated in fiber studies, the benefits of Tempeh as a specific feed have not been clinically validated. However, synbiotic products, probiotic–prebiotic combinations, or gut-health snacks made out of Tempeh have the potential to develop in the population with obesity, inflammatory bowel disease, and metabolic dysregulation.

Not every microbial metabolite can be a good functional bioactive. An example represented by the case of the corrinoid compounds, namely B_12_-like analogues, is quite informative. Initial reports indicated that Tempeh could serve as a source of vitamin B_12_, but recent reports have proven that most of these molecules are inactive analogues, which are not active nutrients in humans^25^.

The contribution of volatiles, predominantly of yeast origin, is also very prominent in the creation of flavor and aroma. Alcohols, esters, and organic acids exhibit minimal direct health effects; however, they significantly impact consumer acceptance and dietary adherence^61^. In the design of functional foods, palatability and sensory quality are crucial for consumer acceptance, thereby rendering yeast metabolism indirectly necessary for the success of Tempeh-based functional products.

A key group of biomarkers of the fermentation process includes bioactive peptides, isoflavone aglycone, phenolic compounds, organic acids, fibrinolytic enzymes, and B-complex vitamins. Bioactive peptides generated through extensive proteolysis by *Rhizopus microsporus*, LAB, and *Bacillus subtilis* increase the abundance of short peptides with angiotensin-converting enzyme (ACE)-inhibitory, antioxidant, anti-inflammatory, and antidiabetic properties. These markers have been tied to cardiovascular and anti-inflammatory activities and are now clearly listed with microbial sources and mechanisms.

**Fig. 4:**
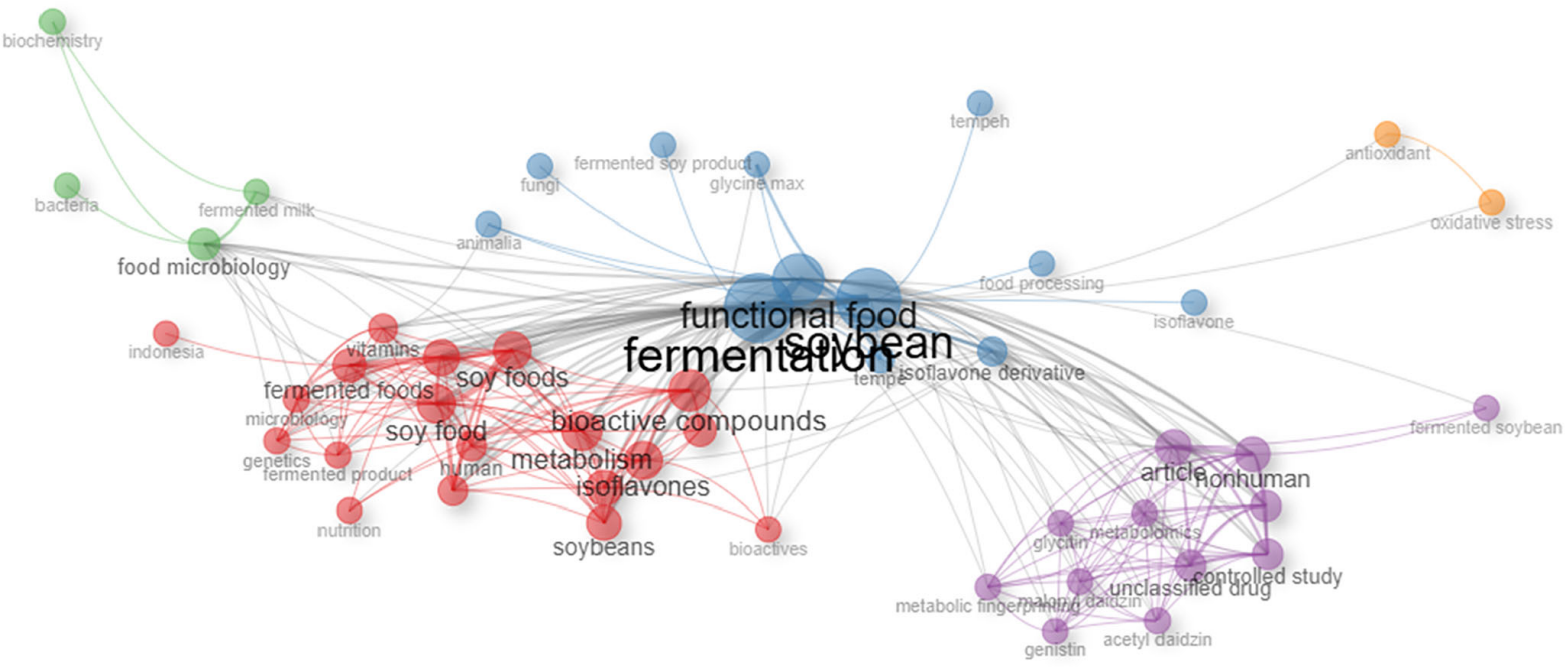
Network map showing correlations between keywords in the articles.

The main keywords in the area of Tempeh as a functional food research by bibliometric analysis. The co-occurrence analysis of keywords, generated using Biblioshiny, highlights the thematic distribution and research focus areas related to the keywords “Tempeh” or “tempe” and “functional food”, as well as the keywords “Tempeh” or “tempe” and “functional food” and “health benefit”. The largest and most prominent term in the network is “fermentation.” This indicates that fermentation is the most important across all publications, and Tempeh is produced by fermentation. Surrounding the term “fermentation”, there are five color-coded clusters. Each of these clusters represents a primary thematic domain within the research, while maintaining interconnectedness across each topic. Figure 4 presents a network map of correlations between keywords in the articles.

Based on Fig. 4, the red cluster is concerned with soy, with research focused on soy foods, soybeans, isoflavones, nutrition, bioactivity, and human health, and on how soy and its bioactive compounds are central to metabolism and diet. In addition to that, speak about microbial ecology and the fermentation basis of Tempeh studies. The keywords that prevail in this cluster are *Rhizopus*, soybean, microorganisms, microbiota, and fermentation process. These words emphasize the role of the *Rhizopus* species as the fundamental fermenters and the role of bacterial consortia, in particular the LAB and *Bacillus* spp., in the formation of the biochemical and safety profile of Tempeh. This group reflects the initial phase of research as it aims at defining the microbial players and the nature of the enzyme activities, as well as how the choice of substrate and conditions of fermentation affect the succession of the microbes.

The blue cluster also takes Tempeh as a fermented product made of soy, which is associated with Glycine max, isoflavone derivatives, food processing, and microbial activity, thus providing insight into the functional food nature of Tempeh as an ingredient with nutritional and cultural uses. In addition to this, comment on the discovery of protease-cleaved peptides, β-glucosidase-mediated isoflavone aglycones, and phytase-mediated mineral bioavailability as conserved bioactivity pathways in soybean and non-soy substrates. The relevance of bioactive peptides and metabolomics in this cluster highlights the increase in the importance of mechanistic research in order to support the findings of Tempeh as a functional food.

The green cluster concentrated on fermented milk and related microbial interactions, bacteria, biochemistry, and microbiology, which denotes research studies on the processes of fermentation in dairy products and their health effects. Moreover, it shows a translational pathway where the biochemical changes occurring in the fermentation process are correlated with the functional ones, including the minimization of oxidative stress, better vascular status, increased mineral absorption, and better digestibility of proteins. The cluster represents the shift of Tempeh research out of the laboratory characterization to the relevance of the research to the field of public health, which is in line with the growing interest in plant-based diets and sustainable protein sources globally.

The purple cluster deals with genistein, with specific isoflavone derivatives, such as genistein, glycitin, daidzein, malonyl daidzein, and acetyl daidzein, and with more advanced methods, including metabolomics and antioxidant profiling, thus representing the biochemical and molecular aspects of the soy research. The fact that these keywords are predominant shows that this cluster is biochemical in nature, and there is a trend whereby more and more research is trying to quantify the variations in the isoflavone concentrations and associate them with functional health outcomes. The fact that metabolomics, antioxidant profiling, and molecular assays have been included in this cluster proves the use of advanced analytical techniques that allow a researcher to trace the presence of isoflavones, their transformation into aglycones, bioavailability, and the antioxidant ability. The purple cluster represents the biochemical and molecular elements of soy and Tempeh studies, in which the traditional gender of health benefits is being validated and mechanistically explained by modern omics-based methods.

The final-colored cluster is the orange cluster that dwells on oxidative stress and targets the health effects of bioactive compounds, especially their antioxidant capabilities in combating oxidative stress, which is an important determinant in the alleviation of chronic diseases. This cluster highlights antioxidant activity and depicts the involvement of protease-derived peptides, isoflavone aglycones, and polyphenolic metabolites in alleviating oxidative damage at the cellular level. The use of vocabulary concerning the antioxidant profiling and stress-response pathways indicates the use of biochemical and cellular assays to support these effects, with the focus on omics methodologies and functional tests to prove the protective role of Tempeh bioactives. The orange cluster indicates the translational nature of the Tempeh research, whereby mechanistic findings on bioactive synthesis are directly linked to their capacity to alleviate oxidative stress and promote long-term health.

Taken together, this scoping review demonstrates that tempeh is a cultural heritage and a field of nutritional science that is interconnected in distinct yet harmonious ways, in which microbial fermentation undergoes profound biochemical and functional transformations. Through the application of omics technologies, including metagenomics, proteomics, and metabolomics, recent studies have begun to elucidate the complex microbial consortia and enzymatic pathways that underlie Tempeh’s enhanced nutritional quality and health-promoting properties. Metagenomics, proteomics, and metabolomics identified the consortium microorganisms (*Rhizopus* sp., LAB, *Bacillus* sp., and yeasts) that carry out enzymatic processes that enrich legumes with nutritional and bioactive compounds, producing bioactive and nutritionally enriched foods. The functional potential of Tempeh is attributable to key markers, including bioactive peptides, isoflavone aglycones, phenolics, vitamins, GABA, and other metabolites.

Nevertheless, to realize the full potential of Tempeh as a functional food for global markets, these findings highlight opportunities to move beyond descriptive research toward integration, standardization, and clinical verification. By connecting omics-based evidence to population health requirements, Tempeh can serve as a viable prototype for functional food innovation and sustainable nutrition, especially plant-based nutrition, cardiovascular health, and micronutrient adequacy. Moreover, the integration of omics provides evidence-based insights through deliberate control of fermentation parameters, offering a realistic pathway to enhance selected bioactive components. Such an approach not only supports the development of improved Tempeh products but also creates opportunities for novel functional ingredients, nutrient-dense, fortified foods, and applications in clinical and population-level nutrition.

## Methods

### Protocol and reporting framework

In this study, the PRISMA 2020 guidelines were followed to remain transparent and reproducible during the process of identifying the studies, screening them, and selecting the suitable ones, as well as during the synthesis.

### Search strategy

An extensive literature search was conducted in Scopus, PubMed, Web of Science, and ScienceDirect to detect omics-based research on Tempeh fermentation published in the period between January 2000 and August 2025. The search query was a combination of controlled terms and free-text words such as “Tempeh,” “*Rhizopus oligosporus*,” “fermentation,” “omics,” “metagenomics,” “proteomics,” “peptidomics,” “metabolomics,” “bioactive peptides,” and “functional foods.” Additional relevant studies were also filtered from the reference lists of the retrieved articles so that they can be covered more widely.

### Eligibility criteria

Two complementary frameworks (SPIDER) (Sample, Phenomenon of Interest, Design, Evaluation, Research type) and PICO (Population, Intervention, Comparison, Outcomes) were used to select the inclusion criteria to certify exhaustive coverage. The SPIDER framework, which focused on the sample (Tempeh or Tempeh-derived products), was used to capture exploratory and qualitative dimensions, encompass the phenomenon of interest (omics-based investigations of fermentation and bioactive compound formation), study design (experimental or analytical), evaluation (reported microbial, enzymatic, metabolite, or bioactive outcomes), and research type (original research articles). Whereas the PICO framework has a different focus, it was applied to guide quantitative and comparative assessments such as defining the population (Tempeh or soybean/legume substrates), intervention (fermentation processes or omics analyses), comparison (pre- vs. post-fermentation, or between strains or conditions), and outcomes (microbial composition, enzymatic pathways, metabolite profiles, and bioactive properties). The eligible listed studies are those that applied at least one omics technique, such as metagenomics, proteomics, metabolomics, or transcriptomics, to Tempeh or Tempeh-based products, reported microbial, enzymatic, metabolite, or bioactive outcomes, and were published in peer-reviewed English-language journals. Both soybean Tempeh and non-soy legume variants were considered. The excluded studies from the review were non-omics studies, reviews, editorials, conference abstracts, non-English publications, and papers not focused explicitly on Tempeh.

### Data extraction and synthesis

The findings were synthesized narratively and categorized thematically into metagenomics, proteomics and peptidomics, metabolomics, and multi-omics integration. This categorization facilitated cross-comparison of microbial pathways, biochemical transformations, and potential health outcomes.

### Bibliometric analysis

**Fig. 5:**
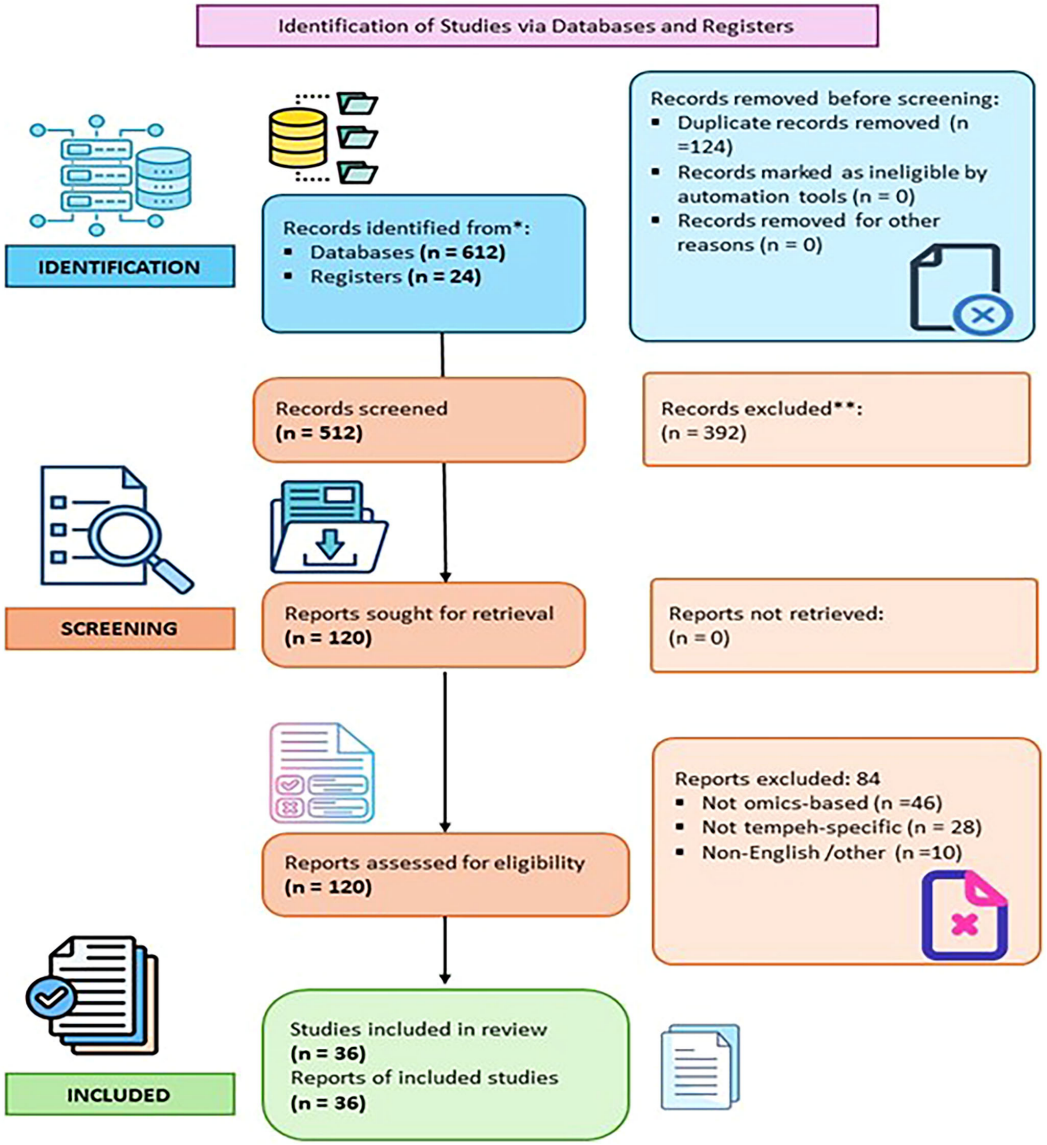
PRISMA Flow Diagram Illustrating The Screening and Selection of Articles Included in This Scoping Review.

To map the trends of publication in the world and co-authorship relationships and clusters of keywords in the topic of Tempeh-omics research, a bibliometric analysis was conducted using VOSviewer. Scopus was used to extract the data on 28 August 2025 in CSV format in Biblioshiny and RIS/BibTeX format in VOSviewer. RStudio version 4.5.1 was used to perform the analysis based on the Bibliometrix/Biblioshiny package, with duplicates removed via DOI-based matching and manual title verification, while VOSviewer Online (Web-based Co-occurrence, Co-authorship, and Citation Mapping Tool) was used to generate network visualizations. It uses ≥5 author keywords, ≥3 documents, and ≥20 citations as thresholds of co-authorship, citation analysis, respectively. To provide the reproducibility of the results, all the raw CSV/RIS files, VOSviewer project files, and parameter settings are provided as supplementary materials. The PRISMA 2020 flow diagram (Fig. 5) shows the process of the study selection. A total number of 636 records were found, of which 512 were found after the elimination of duplicates. Following screening of titles and abstracts, 120 full-text articles were assessed concerning their eligibility, and 36 studies were incorporated in the final review.

## Supplementary information

Supplementary Information

## Data Availability

The data are available on request.

## References

[1] Romulo, A & Surya, R. Tempe: a traditional fermented food of Indonesia and its health benefits. *Int. J. Gastron. Food Sci*. **26**, 10.1016/j.ijgfs.2021.100413 (2021).

[2] Frias, J., Peñas, E. & Martinez-Villaluenga, C. *Fermented Pulses in Nutrition and Health Promotion*. 10.1016/B978-0-12-802309-9.00016-9 (Elsevier Inc., 2017).

[3] Tamam, B. et al. Proteomic study of bioactive peptides from Tempe. *J. Biosci. Bioeng.* **128**, 241–248 (2019). 30930003 10.1016/j.jbiosc.2019.01.019

[4] Astawan, M., Cahyani, A. P. & Wresdiyati, T. Antioxidant activity and isoflavone content of overripe Indonesian tempe. *Food Res.* **7**, 42–50 (2023).

[5] Yarlina, V. P. & Astuti, D. I. Characterization of vitamin B_12_, folate, and isoflavones of soybean Tempeh with the pure isolate Rhizopus oryzae, Rhizopus oligosporus, and Rhizopus stolonifer as functional food,”. *Teknol. Pangan Media Inf. Dan Komun. Ilm. Teknol. Pertan.* **12**, 95–105 (2021).

[6] Herawati, H. et al. Characteristics of GABA (Gamma Amino Butyric Acid), antioxidant and sensory quality of modified Tempeh. *Int. J. Food Prop.* **26**, 3532–3543 (2023).

[7] Sarkar, S., Sha, S.P. & Ghatani, K. Metabolomics of ethnic fermented foods and beverages: understanding new aspects through Omic techniques. *Front. Sustain. Food Syst*. **7**, 10.3389/fsufs.2023.1040567 (2023).

[8] Rezac, S., Kok, C.R., Heermann, M. & Hutkins, R. Fermented foods as a dietary source of live organisms. *Front. Microbiol*. **9**, 10.3389/fmicb.2018.01785 (2018). 10.3389/fmicb.2018.01785 PMC6117398 30197628

[9] Gao, Y. et al. Metabolomics approaches for the comprehensive evaluation of fermented foods: a review. *Foods* **10**, 1–18 (2021). 10.3390/foods10102294 PMC8534989 34681343

[10] Yarlina, V. P., Djali, M., Andoyo, R., Nurmilah, S. & Lani, M. N. Metagenomic insights into enhancing protein content and digestibility in Jack Bean (*Canavalia ensiformis*) Tempeh: unraveling microbial dynamics during fermentation. *Appl. Food Res.* **4**, 100588 (2024).

[11] Marco, M. L. & Tachon, S. Environmental factors influencing the efficacy of probiotic bacteria. *Curr. Opin. Biotechnol.* **24**, 207–213 (2013). 23102489 10.1016/j.copbio.2012.10.002

[12] Yulandi, A., Suwanto, A., Waturangi, D. E. & Wahyudi, A. T. Shotgun metagenomic analysis reveals new insights into bacterial community profiles in Tempeh. *BMC Res. Notes* **13**, 1–7 (2020). 33308279 10.1186/s13104-020-05406-6 PMC7731626

[13] He, M.H. & Howell, K.S. Vitamin-B_12_ enrichment in Tempeh by co-culture with Propionibacterium freudenreichii during fermentation. Preprint at 10.1101/2022.11.06.515253 (2022).

[14] Yarlina, V. P., Andoyo, R., Djali, M. & Lani, M. N. Metagenomic analysis for indigenous microbial diversity in soaking process of making Tempeh Jack Beans (*Canavalia Ensiformis*). *Curr. Res. Nutr. Food Sci.* **10**, 620–632 (2022).

[15] Butowski, C. F., Dixit, Y., Reis, M. M. & Mu, C. Metatranscriptomics for understanding the microbiome in food and nutrition science. *Metabolites* **15**, 10.3390/metabo15030185. (2025). 10.3390/metabo15030185 PMC11943699 40137150

[16] Kustyawati, M. E., Murhadi, S., Rizal, S. & Astuti, P. “Vitamin B_12_ production in soybean fermentation for Tempeh. *AIMS Agric. Food* **5**, 262–271 (2020).

[17] Ríos-Covián, D. et al. Intestinal short chain fatty acids and their link with diet and human health. *Front. Microbiol.* **7**, 1–9 (2016). 26925050 10.3389/fmicb.2016.00185 PMC4756104

[18] Wicaksono, W. A. et al. Traditionally produced Tempeh harbors more diverse bacteria with more putative health-promoting properties than industrially produced Tempeh. *Food Res. Int.* **196**, 115030 (2024). 39614549 10.1016/j.foodres.2024.115030

[19] Astuti, T., Sitompul, M. & Faadhilanisyah, A. Tempeh consumption patterns in Indonesian family and contribution to nutritional adequacy. *AcTion Aceh Nutr. J.* **8**, 518 (2023).

[20] Nout, M. J. R. & Kiers, J. L. Tempe fermentation, innovation and functionality: update into the third millenium. *J. Appl. Microbiol.* **98**, 789–805 (2005). 15752324 10.1111/j.1365-2672.2004.02471.x

[21] Barus, T., Giovania, G. & Lay, B. W. *Lactic Acid Bacteria* from Tempeh and their ability to acidify soybeans in Tempeh fermentation. *Microbiol. Indones.* **14**, 149–155 (2020).

[22] Nurmilah, S. et al. Exploring microbial dynamics and metabolomic profiling of isoflavone transformation in black and yellow soybean tempe for sustainable functional foods. *Food Chem. Mol. Sci.* **11**, 100279 (2025). 10.1016/j.fochms.2025.100279 PMC12309282 40741085

[23] Yarlina, V. P., Nabilah, F., Djali, M., Andoyo, R. & Lani, M. N. Mold characterization in ‘RAPRIMA’ Tempeh yeast from LIPI and over-fermented Koro Pedang (Jack Beans) Tempeh. *Food Res.* **7**, 125–132 (2023).

[24] Samson, R. A. et al. Phylogeny, identification and nomenclature of the genus Aspergillus. *Stud. Mycol.* **78**, 141–173 (2014). 25492982 10.1016/j.simyco.2014.07.004 PMC4260807

[25] Efriwati, A., Suwanto, A., Rahayu, G. & Nuraida, L. “Population dynamics of yeasts and *Lactic Acid Bacteria* (LAB) during tempeh production,” *HAYATI J. Biosci*. **20**, 57–64 (2013).

[26] Seumahu, C. A., Suwanto, A., Rusmana, I. & Solihin, D. D. Bacterial and fungal communities in tempeh as reveal by amplified ribosomal intergenic sequence analysis. *HAYATI J. Biosci.* **20**, 65–71 (2013).

[27] Watanabe, F., Yabuta, Y., Bito, T. & Teng, F. Vitamin B_12_-containing plant food sources for vegetarians. *Nutrients* **6**, 1861–1873 (2014). 24803097 10.3390/nu6051861 PMC4042564

[28] Watanabe, F. Vitamin B_12_ sources and bioavailability. *Exp. Biol. Med.* **232**, 1266–1274 (2007). 10.3181/0703-MR-67 17959839

[29] Teoh, S.Q. et al. A review on health benefits and processing of Tempeh with outlines on its functional microbes. *Futur. Foods* **9**, 100330 10.1016/j.fufo.2024.100330 (2024).

[30] Şanlier, N., Gökcen, B. B. & Sezgin, A. C. Health benefits of fermented foods. *Crit. Rev. Food Sci. Nutr.* **59**, 506–527 (2019). 28945458 10.1080/10408398.2017.1383355

[31] Handoyo, T. & Morita, N. Structural and functional properties of fermented soybean (Tempeh) by using *Rhizopus oligosporus*. *Int. J. Food Prop.* **9**, 347–355 (2006).

[32] Pangastuti, A. et al. Metagenomic analysis of microbial community in over-fermented Tempeh. *Biodiversitas* **20**, 1106–1114 (2019).

[33] Ridhowati, S., Lestari, S. D., Rendi, M. & Rachmawati, S. H. “Characterization of physicochemical properties and enzymatic digestibility of lotus (*Nelumbo nucifera*) Tempeh through different methods. *Food Res.* **7**, 42–52 (2023).

[34] Fusco, W. et al. Short-chain fatty-acid-producing bacteria: key components of the human gut microbiota. *Nutrients* **15**, 10.3390/nu15092211 (2023). 10.3390/nu15092211 PMC10180739 37432351

[35] Puspitojati, E., Indrati, R., Cahyanto, M. N. & Marsono, Y. Formation of ACE-inhibitory peptides during fermentation of jack bean tempe inoculated by usar Hibiscus tiliaceus leaves starter. *IOP Conf. Ser. Earth Environ. Sci.* **292**, 0–8 (2019).

[36] Purwandari, F. A., Fogliano, V. & Capuano, E. Tempeh fermentation improves the nutritional and functional characteristics of Jack beans (Canavalia ensiformis (L.) DC). *Food Funct.* **15**, 3680–3691 (2024). 38488045 10.1039/d3fo05379b

[37] Chao, S.H. et al. Microbial diversity analysis of fermented mung beans (Lu-Doh-Huang) by using pyrosequencing and culture methods. *PLoS ONE* **8**, 10.1371/journal.pone.0063816 (2013). 10.1371/journal.pone.0063816 PMC3659079 23700436

[38] Kiers, J.L. *Effects of fermented soya bean on digestion, absorption and diarrhoea* 110 (Wageningen Universiteit, 2001).

[39] Monika, S. avitri, Kumar, V., Kumari, A., Angmo, K. & Bhalla, T. C. “Isolation and characterization of *Lactic Acid Bacteria* from traditional pickles of Himachal Pradesh, India. *J. Food Sci. Technol.* **54**, 1945–1952 (2017). 28720951 10.1007/s13197-017-2629-1 PMC5495720

[40] Barus, T. et al. Genetic diversity of Klebsiella spp. isolated from tempe based on enterobacterial repetitive intergenic consensus-polymerase chain reaction (ERIC-PCR). *HAYATI J. Biosci.* **20**, 171–176 (2013).

[41] Kuligowski, M., Pawłowska, K. & Jasińska-kuligowska, I. Isoflavone composition, polyphenols content and antioxidative activity of soybean seeds during Tempeh fermentation. *CyTA J. Food* **15**, 27–33 (2017).

[42] Kwon, D. Y., Daily, J. W., Kim, H. J. & Park, S. Antidiabetic effects of fermented soybean products on type 2 diabetes. *Nutr. Res.* **30**, 1–13 (2010). 20116654 10.1016/j.nutres.2009.11.004

[43] Messina, M. Soy and health update: evaluation of the clinical and epidemiologic literature. *Nutrients* **8**, 10.3390/nu8120754 (2016). 10.3390/nu8120754 PMC5188409 27886135

[44] Javed, Z. et al. Genistein as a regulator of signaling pathways and microRNAs in different types of cancers. *Cancer Cell Int.* **21**, 1–12 (2021). 34289845 10.1186/s12935-021-02091-8 PMC8296701

[45] Naeem, H. et al. Anticancer perspectives of genistein: a comprehensive review. *Int. J. Food Prop.* **26**, 3305–3341 (2023).

[46] Messina, M. Insights gained from 20 years of soy research. *J. Nutr.* **140**, 2289–2295 (2010). 10.3945/jn.110.124107 20980639

[47] Mackay, G. M. et al. Tryptophan metabolism and oxidative stress in patients with chronic brain injury. *Eur. J. Neurol.* **13**, 30–42 (2006). 16420391 10.1111/j.1468-1331.2006.01220.x

[48] Szerszunowicz, I. & Kozicki, S. “Plant-derived proteins and peptides as potential immunomodulators. *Molecules* **29**, 10.3390/molecules29010209 (2024). 10.3390/molecules29010209 PMC10780438 38202792

[49] Granato, D. et al. Functional foods: product development, technological trends, efficacy testing, and safety. *Annu. Rev. Food Sci. Technol.* **11**, 93–118 (2020). 31905019 10.1146/annurev-food-032519-051708

[50] Frias, J., Young, S. S., Martínez-Villaluenga, C., De Mejia, E. G. & Vidal-Valverde, C. Immunoreactivity and amino acid content of fermented soybean products. *J. Agric. Food Chem.* **56**, 99–105 (2008). 18072744 10.1021/jf072177j

[51] Danial, A. M., Peng, K. S. & Long, K. Enrichment of Mung Bean with L-DOPA, GABA, essential amino acids via controlled biofermentation strategy. *Int. J. Biotechnol. Wellness Ind.* **4**, 114–122 (2016).

[52] Bahar, A. & Witono, Y. Process optimization of Tempeh protein isolate from Soybean (*Glycine max* Merr) and Cowpea (*Vigna unguiculata*) mixture. *Int. J. Adv. Sci. Eng. Inf. Technol.* **5**, 139–143 (2015).

[53] Leblanc, J. G. et al. B-Group vitamin production by *Lactic Acid Bacteria* - current knowledge and potential applications. *J. Appl. Microbiol.* **111**, 1297–1309 (2011). 21933312 10.1111/j.1365-2672.2011.05157.x

[54] Masuda, M. et al. Production potency of folate, Vitamin B_12_, and thiamine by *Lactic Acid Bacteria* isolated from Japanese pickles. *Biosci. Biotechnol. Biochem.* **76**, 2061–2067 (2012). 23132566 10.1271/bbb.120414

[55] Egounlety, M. & Aworh, O. Effect of soaking, dehulling, cooking and fermentation with Rhizopus oligosporus on the oligosaccharides, trypsin inhibitor, phytic acid and tannins of soybean (GlycinemaxMerr.), cowpea (Vigna unguiculata L. Walp) and ground bean (Macrotyloma geocarpa Ha. *J. Food Eng.* **56**, 249–254 (2003).

[56] Narad, P. & Kirthanashri, S. V. Introduction to Omics. in *Omics approaches, technologies and applications: integrative approaches for understanding OMICS data* 1–10 (Springer Nature Singapore, 2018).

[57] Yoshii, H. et al. Antihypertensive effect of ACE inhibitory oligopeptides from chicken egg yolks. *Comp. Biochem. Physiol. C Pharmacol. Toxicol. Endocrinol.* **128**, 27–33 (2001). 10.1016/s1532-0456(00)00172-1 11166671

[58] Diana, M., Quílez, J. & Rafecas, M. Gamma-aminobutyric acid as a bioactive compound in foods: a review. *J. Funct. Foods* **10**, 407–420 (2014).

[59] Nkhata, S.G., Ayua, E., Kamau, E.H. & Shingiro, J.B. Fermentation and germination improve nutritional value of cereals and legumes through activation of endogenous enzymes. *Food Sci. Nutr*. **6**, 2446–2458 (2018) . 10.1002/fsn3.846 PMC6261201 30510746

[60] Steinkraus, K. H. Fermentations in world food processing. *Compr. Rev. Food Sci. Food Saf.* **1**, 23–32 (2002). 33451246 10.1111/j.1541-4337.2002.tb00004.x

[61] Sumathy, J. “A study on the enterobacteriaceae pathogen klebsiella pneumoniae isolated from sewage and drinking water environment. *IOSR J. Biotechnol. Biochem.* **4**, 1–5 (2018).

[62] Cui, Y., Miao, K., Niyaphorn, S. & Qu, X. Production of gamma-aminobutyric acid from *Lactic Acid Bacteria*: a systematic review. *Int. J. Mol. Sci.* **21**, 1–21 (2020). 10.3390/ijms21030995 PMC7037312 32028587

[63] Moa, H. et al. “Effect of soybean processing on content and bioaccessibility of folate, vitamin B_12_ and isoflavones in tofu and tempe. *Food Chem.* **141**, 2418–2425 (2013). 23870976 10.1016/j.foodchem.2013.05.017

[64] Teixeira-Guedes, C., Sánchez-Moya, T., Pereira-Wilson, C., Ros-Berruezo, G. & López-Nicolás, R. In vitro modulation of gut microbiota and metabolism by cooked cowpea and black bean. *Foods* **9**, 10.3390/foods9070861 (2020). 10.3390/foods9070861 PMC7404724 32630276

[65] Poernomo, A. T. Isnaeni, and Purwanto, “Aktivitas In*vitro* Enzim Fibrinolitik Ekstrak Tempe Hasil Fermentasi Rhizopus Oligosporus ATCC 6010 Pada Substrat Kedelai Hitam. *Berk. Ilm. Kim. Farm.* **4**, 18–24 (2014).

[66] Gibbs, B. F., Zougman, A., Masse, R. & Mulligan, C. Production and characterization of bioactive peptides from soy hydrolysate and soy-fermented food. *Food Res. Int.* **37**, 123–131 (2004).

[67] Barus, T., Suwanto, A. & Agustina, W. Metagenomic analysis of bacterial diversity in tempe using terminal restriction fragment length polymorphism (T-RFLP) Technique. *Biota* **15**, 80–273 (2010).

[68] Astawan, M., Mardhiyyah, Y. S. & Wijaya, C. H. Potential of bioactive components in tempe for the treatment of obesity. *J. Gizi dan Pangan* **13**, 79–86 (2018). pp.

[69] Duliński, R. & Starzyńska-Janiszewska, A. “Content and in vitro bioavailability of selected B vitamins and myo-inositol in spelt wheat (Triticum spelta L.) subjected to solid-state fermentation. *J. Food Nutr. Res.* **59**, 1–6 (2020).

[70] Maryati, Y., Susilowati, A., Melanie, H. & Lotulung, P.D. Fermentation of soybean (*Glycine* max (L.) Merr.) using mix inocula of Rhizopus sp. and *Saccharomyces cerevisiae* for an alternative source of folic acid. *IOP Conf. Ser. Mater. Sci. Eng*. **536**, 10.1088/1757-899X/536/1/012124. (2019).

[71] Agustina, R.K., Dieny, E.F. & Rustanti, N. Antioxidant activity and soluble protein content of tempeh gembus hydrolysate. *Hiroshima J. Med. Sci*. **67**, 1–7 (2018).

[72] Wang, R. et al. Preparation of bioactive peptides with antidiabetic, antihypertensive, and antioxidant activities and identification of α-glucosidase inhibitory peptides from soy protein. *Food Sci. Nutr.* **7**, 1848–1856 (2019). 31139399 10.1002/fsn3.1038 PMC6526634

[73] Sybesma, W., Starrenburg, M., Tijsseling, L., Hoefnagel, M. H. N. & Hugenholtz, J. Effects of cultivation conditions on folate production by *Lactic Acid Bacteria*. *Appl. Environ. Microbiol.* **69**, 4542–4548 (2003). 12902240 10.1128/AEM.69.8.4542-4548.2003 PMC169137

[74] Rizal, S., Murhadi, M., Kustyawati, M. E. & Hasanudin, U. Growth optimization of saccharomyces cerevisiae and rhizopus oligosporus during fermentation to produce Tempeh with high β-glucan content. *Biodiversitas* **21**, 2667–2673 (2020).

[75] Prativi, M. B. N. et al. “Metabolite changes in Indonesian Tempe production from raw soybeans to over-fermented Tempe. *Metabolites* **13**, 300 (2023). 36837919 10.3390/metabo13020300 PMC9958738

[76] Rong, P. et al. Untargeted metabolomics analysis of non-volatile metabolites and dynamic changes of antioxidant capacity in Douchi with edible mushroom. *Food Chem.* **431**, 137066 (2024). 37572484 10.1016/j.foodchem.2023.137066

[77] Mei Feng, X., Ostenfeld Larsen, T. & Schnürer, J. Production of volatile compounds by Rhizopus oligosporus during soybean and barley Tempeh fermentation. *Int. J. Food Microbiol.* **113**, 133–141 (2007). 16889859 10.1016/j.ijfoodmicro.2006.06.025

[78] Kadar, A. D., Astawan, M., Putri, S. P. & Fukusaki, E. Metabolomics-based study of the effect of raw materials on the end product of tempe—an Indonesian fermented soybean. *Metabolites* **10**, 1–11 (2020). 10.3390/metabo10090367 PMC7569771 32932879

[79] Panchaud, A., Affolter, M. & Kussmann, M. Mass spectrometry for nutritional peptidomics: how to analyze food bioactives and their health effects. *J. Proteomics* **75**, 3546–3559 (2012). 22227401 10.1016/j.jprot.2011.12.022

[80] Nemec, A. et al. Taxonomy of haemolytic and/or proteolytic strains of the genus *Acinetobacter* with the proposal of *Acinetobacter courvalinii* sp. Nov. (genomic species 14 sensu Bouvet and Jeanjean), Acinetobacter dispersus sp. nov. (genomic species 17), Acinetobacter modes,”. *Int. J. Syst. Evol. Microbiol.* **66**, 1673–1685 (2016). 26822020 10.1099/ijsem.0.000932

[81] Olm, M. R., Brown, C. T., Brooks, B. & Banfield, J. F. “dRep : a tool for fast and accurate genomic comparisons that enables improved genome recovery from metagenomes through de-replication. *ISME J.* **11**, 2864–2868 (2017). 10.1038/ismej.2017.126 PMC5702732 28742071

[82] Prakash, J. et al. Analysis of meta-transcriptomics and identification of genes linked to bioactive peptides and vitamins in Indonesian tempe. *Food Res. Int.* **202**, 115757 (2025). 39967073 10.1016/j.foodres.2025.115757

[83] Akinyemi, O. Integrated multi-omics approach to investigate the microbiome of Tempeh, a traditional Indonesian fermented food. (2024).

[84] Kuczynski, J. et al. Using QIIME to analyze 16S rRNA gene sequences from microbial communities. *Curr. Protoc. Bioinform*. **36**, 10.1002/0471250953.bi1007s36 (2011). 10.1002/0471250953.bi1007s36 PMC3249058 22161565

[85] Pruesse, E. et al. SILVA: a comprehensive online resource for quality checked and aligned ribosomal RNA sequence data compatible with ARB. *Nucleic Acids Res.* **35**, 7188–7196 (2007). 17947321 10.1093/nar/gkm864 PMC2175337

[86] Yulandi, Y., Suwanto, A., Waturangi, D.E. & Wahyudi, A.T. Shotgun metagenomic analysis reveals new insights into bacterial community profiles in Tempeh. *BMC Res.* **13**, 10.1186/s13104-020-05406-6 (2020). 10.1186/s13104-020-05406-6 PMC7731626 33308279

